# Heavy

**Published:** 1883-04-20

**Authors:** 


### *HEAVY.*

Dr. DaCosta is reported as saying (Med. Herald) "If one has not too much to do he writes a short paper on *Phthisis.* If one has little to do he writes a long paper on *Phthisis.* If one has nothing to do he writes a book on *Phthisis.*" He further states that "Gynecologists, as a rule. part their hair and their names in the middle, and never die until they have invented pessaries and speculums innumerable."

